# Supplementary material for: Content-rich biological network constructed by mining PubMed abstracts
Source: BMC Bioinformatics. 2004 Oct 8;5:147. doi: 10.1186/1471-2105-5-147 (PMC528731; doi:10.1186/1471-2105-5-147)
Supplement: Additional File 2 — The original results of the above study (non-essential files are deleted to keep the file size under the limit set by BMC bioinformatics). [file 1471-2105-5-147-S2.bz2 › chilibotAdditionalFile2/dip50/275ID1922387E1095/html/MAPK10_JUN.html]

 


 **MAPK10** and **JUN** 
  
Found 55 abstracts in PubMed, retrieved 50.  
 

 What does Google say? 
 PDF only 
| .edu only 

---

**Interactive relationship** (e.g. stimulation, inhibition, etc)

**Inhibitory relationship**- Second, we show that HNE induced c  **Jun**  phosphorylation and c  **jun**  induction are attenuated in JNK3  [ **MAPK10** ]  deficient neurons.  Ref: 12424734 J Neurosci Res, 2002
- In contrast, NGF deprivation induced increases in the levels of phosphorylated c  **Jun** , c  **jun** , and apoptosis are each inhibited in JNK3  [ **MAPK10** ]  deficient mice.  Ref: 11461965 J Neurochem, 2001
**Stimulatory relationship**- Herein, we provide cDNA microarray evidence that METH administration caused the induction of c  **Jun**  and of other members involved in the pathway leading to c  **Jun**  activation stress activated protein kinase  **Jun**  N terminal kinase JNK3  [ **MAPK10** ] , Crk associated substrate Cas and c Src after environmental stresses or cytokine stimulation.  Ref: 11961130 Mol Pharmacol, 2002
- Here, we show that cdk5 directly phosphorylates c  **Jun**  N terminal kinase 3  [ **MAPK10** ]  JNK3  [ **MAPK10** ]  on Thr131 and inhibits its kinase activity, leading to reduced c  **Jun**  phosphorylation.  Ref: 11823425 EMBO J, 2002
**Neutral relationship**- JNK3  [ **MAPK10** ]  contributes to c  **jun**  induction and apoptosis in 4 hydroxynonenal treated sympathetic neurons.  Ref: 12424734 J Neurosci Res, 2002
- JNK3  [ **MAPK10** ]  contributes to c  **Jun**  activation and apoptosis but not oxidative stress in nerve growth factor deprived sympathetic neurons.  Ref: 11461965 J Neurochem, 2001
- c  **Jun**  N terminal kinase 3  [ **MAPK10** ]  JNK3  [ **MAPK10** ]  stress activated protein kinase beta SAPKbeta binds and phosphorylates the neuronal microtubule regulator SCG10.  Ref: 11718727 FEBS Lett, 2001
- Particularly, JNK3  [ **MAPK10** ]  is considered to be a degenerative enzyme with c  **Jun**  as a relevant substrate.  Ref: 12401814 J Biol Chem, 2003
- Overall, these results indicate that JNK3  [ **MAPK10** ]  plays a critical role in activation of c  **Jun**  and apoptosis in a classic model of cell autonomous programmed neuron death.  Ref: 11461965 J Neurochem, 2001

**Non-interactive relationship** (e.g. studied together, co-existance, homology, etc.)

- We have identified a novel  **Jun**  N terminal kinase JNK binding protein, termed JNKBP1, and examined its binding affinity for JNK1, JNK2, JNK3  [ **MAPK10** ] , and extracellular signal regulated kinase 2 ERK2 in COS 7 cells.  Ref: 10471813 FEBS Lett, 1999
- Kainate receptor glutamate receptor 6 GluR6 subunit deficient and c  **Jun**  N terminal kinase 3  [ **MAPK10** ]  JNK3  [ **MAPK10** ]  null mice share similar phenotypes including resistance to kainite induced epileptic seizures and neuronal toxicity Yang, D.  Ref: 11152698 J Biol Chem, 2001
- In summary, these results indicate that JNK3  [ **MAPK10** ]  plays a critical role in HNE induced c  **Jun**  activation and apoptosis in sympathetic neurons.  Ref: 12424734 J Neurosci Res, 2002
- Both corresponded to the c  **Jun**  NH2 terminal kinase JNK 3  [ **MAPK10** ] .  Ref: 11322657 J Hum Genet, 2001
- The mitogen activated kinase activating death domain protein MADD that is differentially expressed in neoplastic vs. normal cells DENN was identified as a substrate for c  **Jun**  N terminal kinase 3  [ **MAPK10** ] , the first demonstration of such an activity for this stress activated kinase that is predominantly expressed in the brain.  Ref: 9482930 Proc Natl Acad Sci U S A, 1998
- A single c  **Jun**  N terminal kinase isoform JNK3  [ **MAPK10** ]  p54 is an effector in both neuronal differentiation and cell death.  Ref: 12401814 J Biol Chem, 2003
- c  **Jun**  N terminal kinase 3  [ **MAPK10** ]  JNK3  [ **MAPK10** ] , the only neural specific isoform, may play an important role in excitotoxicity and neuronal injury.  Ref: 12767482 Neurosci Res, 2003
- Moreover, cdk5 deficient cultured cortical neurons exhibit increased sensitivity to apoptotic stimuli, as well as elevated JNK3  [ **MAPK10** ]  activity and c  **Jun**  phosphorylation.  Ref: 11823425 EMBO J, 2002
- These may include the extracellular regulated protein kinases ERK1 and ERK2, the c  **Jun**  N terminal kinases JNK1, JNK2, and JNK3  [ **MAPK10** ] , and one or more isoenzymes of protein kinase C.  Ref: 10900180 J Mol Cell Cardiol, 2000
- To evaluate directly the apoptotic role of the JNK isoform that is selectively expressed in neurons, JNK3  [ **MAPK10** ] , we isolated sympathetic neurons from JNK3  [ **MAPK10** ]  deficient mice and quantified nerve growth factor NGF deprivation induced neuronal death, oxidative stress, c  **Jun**  phosphorylation, and c  **jun**  induction.  Ref: 11461965 J Neurochem, 2001
- A significant advance in the understanding of how GPCRs activate MAPK cascades is the discovery that beta arrestin, a protein well known for its roles in both receptor desensitization and internalization, serves as a scaffolding protein for at least two GPCR stimulated MAPK cascades, the extracellular signal regulated kinase ERK cascade and the c  **jun**  N terminal kinase 3  [ **MAPK10** ]  JNK3  [ **MAPK10** ]  cascade.  Ref: 11313899 Oncogene, 2001
- Although previous evidences indicate that JNK3  [ **MAPK10** ]  is required for neuronal death under certain conditions, we show that JNK3  [ **MAPK10** ]  deficiency only partially mediates c  **Jun**  phosphorylation and its deficiency does not protect neurons from death.  Ref: 12091388 J Biol Chem, 2002
- We identified c  **Jun**  amino terminal kinase 3 JNK3  [ **MAPK10** ]  as a binding partner of beta arrestin 2 using a yeast two hybrid screen and by coimmunoprecipitation from mouse brain extracts or cotransfected COS 7 cells.  Ref: 11090355 Science, 2000
- When characterizing the 5 flanking region of the c  **Jun**  NH2 terminal kinase 3 gene at 4q21 22, where frequent allelic losses and loss of expression had been detected in patients with brain tumors and hepatocellular carcinomas, we discovered that the Fas associated phosphatase 1 gene was located only 633 bp upstream from JNK3  [ **MAPK10** ]  in a head to head orientation.  Ref: 12436199 J Hum Genet, 2002
- The c  **Jun**  NH2 terminal kinase3 JNK3  [ **MAPK10** ]  gene genomic structure, chromosomal assignment, and loss of expression in brain tumors.  Ref: 11322657 J Hum Genet, 2001
- Here, we have identified a novel mouse protein, termed  **Jun**  N terminal protein kinase JNK stress activated protein kinase associated protein 1 JSAP1, by a yeast two hybrid screen, using JNK3  [ **MAPK10** ]  MAPK as the bait.  Ref: 10523642 Mol Cell Biol, 1999
- Here, we report that p75 activates Rac GTPase, which in turn activates c  **jun**  N terminal kinase JNK, including an injury specific JNK3  [ **MAPK10** ] , in an NGF dependent manner.  Ref: 11756498 J Neurosci, 2002
- Cyclin dependent kinase 5 prevents neuronal apoptosis by negative regulation of c  **Jun**  N terminal kinase 3  [ **MAPK10** ] .  Ref: 11823425 EMBO J, 2002
- Recently, we identified the c  **Jun**  N terminal kinase 3  [ **MAPK10** ]  JNK3  [ **MAPK10** ]  as a beta arrestin2 interacting protein in yeast two hybrid and co immunoprecipitation studies.  Ref: 11356842 J Biol Chem, 2001
